# Transmural Ultrasound Imaging of Thermal Lesion and Action Potential Changes in Perfused Canine Cardiac Wedge Preparations by High Intensity Focused Ultrasound Ablation

**DOI:** 10.1371/journal.pone.0082689

**Published:** 2013-12-12

**Authors:** Ziqi Wu, Madhu S. R. Gudur, Cheri X. Deng

**Affiliations:** Department of Biomedical Engineering, University of Michigan, Ann Arbor, Michigan, United States of America; University of Minnesota, United States of America

## Abstract

Intra-procedural imaging is important for guiding cardiac arrhythmia ablation. It is difficult to obtain intra-procedural correlation of thermal lesion formation with action potential (AP) changes in the transmural plane during ablation. This study tested parametric ultrasound imaging for transmural imaging of lesion and AP changes in high intensity focused ultrasound (HIFU) ablation using coronary perfused canine ventricular wedge preparations (n = 13). The preparations were paced from epi/endocardial surfaces and subjected to HIFU application (3.5 MHz, 11 Hz pulse-repetition-frequency, 70% duty cycle, duration 4 s, 3500 W/cm^2^), during which simultaneous optical mapping (1 kframes/s) using di-4-ANEPPS and ultrasound imaging (30 MHz) of the same transmural surface of the wedge were performed. Spatiotemporally correlated AP measurements and ultrasound imaging allowed quantification of the reduction of AP amplitude (APA), shortening of AP duration at 50% repolarization, AP triangulation, decrease of optical AP rise, and change of conduction velocity along tissue depth direction within and surrounding HIFU lesions. The threshold of irreversible change in APA correlating to lesions was determined to be 43±1% with a receiver operating characteristic (ROC) area under curve (AUC) of 0.96±0.01 (n = 13). Ultrasound imaging parameters such as integrated backscatter, Rayleigh (*α*) and log-normal (σ) parameters, cumulative extrema of σ were tested, with the cumulative extrema of σ performing the best in detecting lesion (ROC AUC 0.89±0.01, n = 13) and change of APA (ROC AUC 0.79±0.03, n = 13). In conclusion, characteristic tissue and AP changes in HIFU ablation were identified and spatiotemporally correlated using optical mapping and ultrasound imaging. Parametric ultrasound imaging using cumulative extrema of σ can detect HIFU lesion and APA reduction.

## Introduction

Cardiac arrhythmias are common cardiac conditions occurring in patients with or without overt cardiac diseases. Ablation using various energies, e.g. radiofrequency, cryo, high intensity focused-ultrasound (HIFU), has increasingly become an important treatment option [1], [2]. However, ablation efficacy is often compromised by recurrence of arrhythmic episodes after initial procedure. For example, 20 – 40% of patients experience recurrent atrial fibrillation (AF) and will need repeated ablation procedures [3]. This is likely due to incomplete tissue necrosis, ablation gaps, and electrical reconnection from tissue healing [4], [5], [6], [7]. Intra-operative electrogram and electroanatomical voltage mapping have been used to confirm success of ablation clinically, but it is difficult to relate such surface measurements to tissue changes in a transmural plane precisely due to the lack of effective imaging technique for detecting lesion and action potential (AP) changes along the tissue depth direction.

Various imaging techniques have been developed for assessing thermal lesion during ablation. Magnetic resonance imaging (MRI) [8], intracardiac echocardiography (ICE) [9], and fluoroscopy, commonly used to identify anatomic structures to guide placement of ablation catheter, have not provided sufficient intra-operative assessment of tissue physical and electrophysiological (EP) changes. Measurements such as tissue electrical impedance [10], ablation temperature [11], and tissue-catheter contact force [12] provide useful feedback for energy titration and prediction of lesion volume, but accuracies of lesion estimation are suboptimal. Direct tissue surface visualization using endoscopy during pulmonary vein isolation [13] cannot provide complete lesion assessment in a transmural plane along the tissue depth direction. Optical coherence tomography, with superior imaging resolution, is limited by imaging tissue depth only up to 2 mm [14].

As ultrasound imaging has the unique capability to “see” through soft tissue layers, various advanced ultrasound imaging techniques have been exploited to improve its capability for real time monitoring of lesion formation into subsurface tissue layers. For examples, Wright *et al.* showed a novel M-mode ultrasound imaging system integrated into a radiofrequency ablation (RFA) catheter for direct visualization of transmural lesion formation in real time [15], demonstrating an advantageous approach for image-guided procedures. Kolios *et al.* demonstrated high frequency ultrasound backscatter signals can be used to effectively detect apoptotic changes in cell nucleus using spectral analysis method [16], indicating changes in the nucleus, such as apoptotic, necrosis or other changes, many produce detectable signals for high frequency ultrasound imaging for monitoring events reflective of cellular level changes. We have previously shown that high frequency ultrasound imaging at 55 MHz combined with fast frame rate M-mode (1 kHz) and short time B-mode (70 – 130 frames/s) can image HIFU lesion and gas body formation [17], [18] with high accuracy and resolution by tracking the temporal history of ultrasound integrated backscatter (IBS) and frame-to-frame echo decorrelation. ICE-based acoustic radiation force impulse (ARFI) imaging also provided improved performance in identifying lesion boundaries and dimensions in RFA lesion lines [19], although motion artifacts sometimes led to false classification of lesions with low imaging frame rate (<4 frames/min).

Electroanatomical mapping combined with pacing technique is used clinically for validating ectopic foci isolation after ablation, however, the technique is based on surface measurement thus it is difficult to directly relate such measurements to transmural tissue status in a three-dimensional (3D) space. Given that cellular repolarization varies with the depth of myocardium across ventricular wall due to heterogeneous excitation-contraction coupling [20], it is unclear whether these heterogeneous cellular AP characteristics in different transmural tissue layers influenced by ablation are reflected precisely in surface measurements. Using microelectrode recording at isolated locations within myocardium, Wood *et al.* showed changes of AP amplitude, AP duration, and upstroke velocity surrounding an RF lesion across epi-, mid, and endocardium [21], however, a complete spatiotemporal evaluation of EP changes across the depth of the myocardium in a transmural plane during and after lesion formation has never been done previously.

Development of imaging for accurate lesion detection and action potential assessment in a transmural plane can be highly beneficial for ablation guidance. Previously, we have demonstrated monitoring of HIFU generated cardiac EP changes on intact rabbit heart using optical mapping [22]. In this study, in order to investigate the spatiotemporal changes of tissue and correlated with AP changes in a transmural plane along the tissue depth direction during HIFU ablation, we used perfused canine heart wedge preparations as a model system which enabled simultaneous ultrasound imaging and optical mapping on the same transmural plane during HIFU ablation. Although the wedge preparations are not necessarily the best model representing an intact heart *in vivo*, the model system permitted high spatiotemporal resolution measurement of EP changes during HIFU ablation using optical mapping that can be correlated with ultrasound imaging of the same tissue plane, which cannot be achieved with an intact heart either *in vitro* or *in vivo*. These measurements allowed characterization of the EP changes induced by HIFU ablation, and by correlating with lesion histology, we tested parametric ultrasound imaging for detecting lesion and AP changes across the ventricular depth direction during HIFU ablation.

## Materials and Methods

### Ethics statement

Dogs were obtained from a class B dealer (Kenneth Schroeder, Hodgins Kennels, Inc., R&R Research, USDA license#: 41-B-0017, 34-B-0002, 34-B-0001 respectively). Animal use was approved by University Committee on Use and Care of Animals at University of Michigan (Protocol ID# PRO00001220). The University of Michigan is fully accredited with the American Association for Accreditation of Laboratory Animal Care International (AAALAC), and care of the animals adhered to the standards in The Eighth Edition of the *Guide for the Care and Use of Laboratory Animals* (NRC 2011).

### Perfused canine wedge preparations

**Figure 1:**
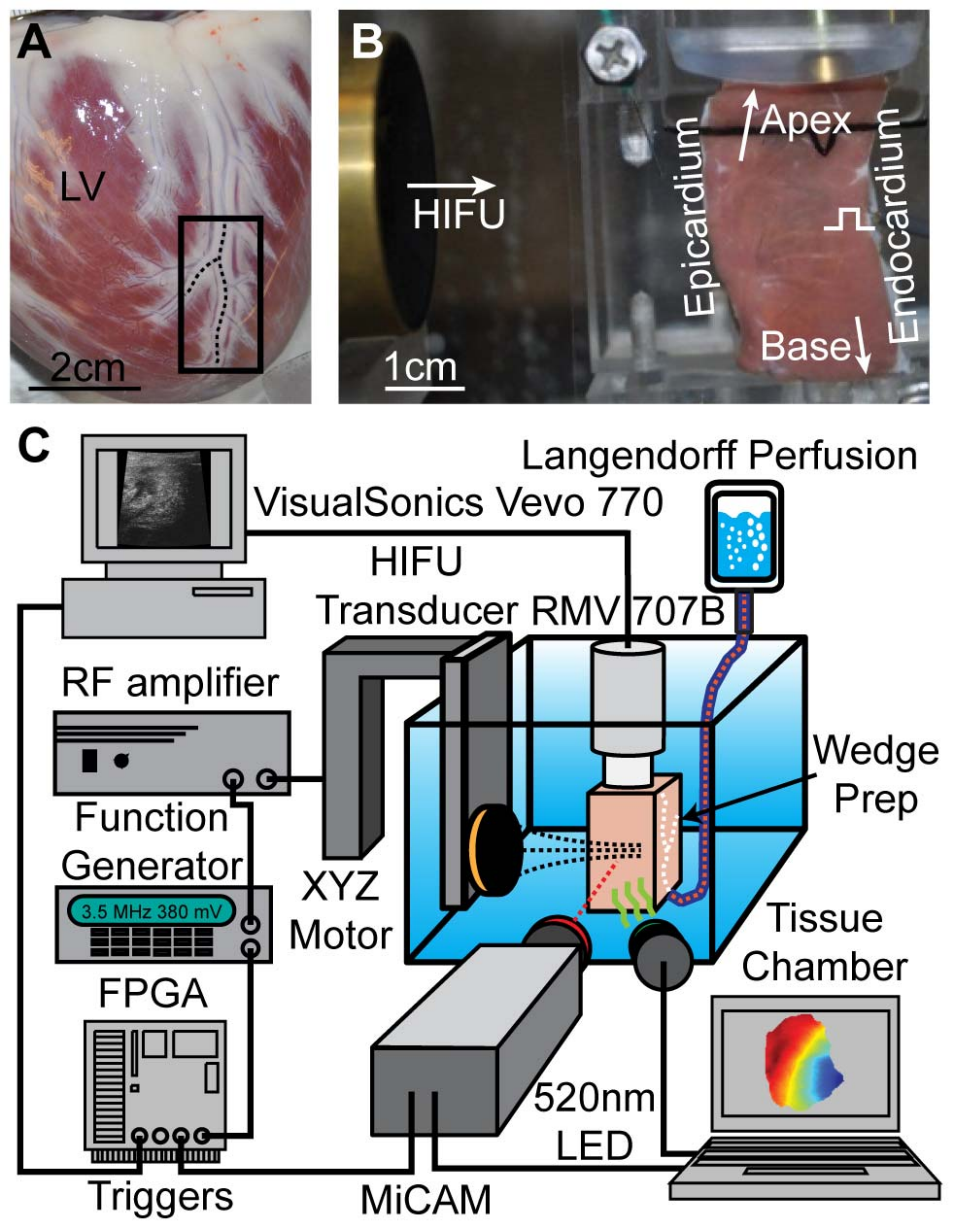
Canine left ventricular wedge and experimental setup with optical mapping, ultrasound imaging, and HIFU ablation. (A) Photograph of a canine heart immersed in cardioplegia before wedge preparation. Black box indicates the tissue region used. Dash lines highlight marginal coronary arteries. (B) Photograph of the transmural surface of a wedge with HIFU transducer at the left and ultrasound imaging in the same plane from the top. In thirteen dog experiments, HIFU was applied from either endocardial (n = 8) or epicardial (n = 5) side. (C) Schematic experimental setup. The HIFU transducer was mounted on a high precision XYZ motor stage. Imaging and ablation were synchronized via a custom programmed FPGA circuit.

A total of 13 mongrel dogs (25 – 40 kg) of either sex were used in this study. Anesthesia was performed with intravenous administration of acepromazine (1 mg/kg, maximum 3 mg total) and propofol (2 – 8 mg/kg), and maintained by inhalation of 1 – 2% isoflurane to alleviate the discomfort. Euthanasia was achieved with overdose sodium pentobarbital injection (140 – 160 mg/kg) introvenously. The heart was then removed following a median sternotomy and the transmural wedges of canine ventricular wall were isolated as described before [23]. Briefly, freshly harvested canine heart was immediately aortic rinsed and arrested with a 4°C cardioplegic solution. A wedge (∼4×1.5×1.5 cm^3^) with smooth transmural surfaces was dissected from the anterolateral or posterior-lateral free wall of either left ventricle (LV) or right ventricle (RV) supplied by left anterior descending (LAD) coronary arteries or marginal right coronary arteries (RCA) (Figure 1A). A flexible plastic cannula was inserted into the artery from the base, and major arterial leaks were quickly (<10 mins) ligated with suture. Perfusion was validated by injection of Methylene Blue dye (Sigma, St. Louis, MO), and poorly perfused wedges were discarded. The wedge was placed onto a perfusion apparatus, and perfused with oxygenated Tyrode's solution [24] (pH = 7.35±0.05; 37°C; 95% O_2_/5% CO_2_) at constant arterial pressure (50 – 70 mmHg). The preparation was paced from either endocardium or epicardium at a cycle length (CL) of 1000 ms until a stable Q-T interval was reached on ECG (30 – 40 mins). The ECG and arterial pressure were continuously monitored (PowerLab 26T, AD Instruments) via two floating electrodes. After adding excitation-contraction decoupler, 2, 3-butanedione monoxime (BDM, 15 mM; Fisher Scientific) into the perfusate, the wedge was stained with a voltage-sensitive dye di-4-ANEPPS (15 µM; Invitrogen).

### Experimental setup

As illustrated in Figure 1B – C, the transmural surface of the wedge was gently pushed against a flat polycarbonate film/screen (acoustic transparent) to maintain tissue shape (Figure 1B). A concave single element HIFU transducer (3.5 MHz, SU-102, Sonic Concepts), driven by a signal generator (33250A, Agilent Technologies) and a 75W power amplifier (75A250, Amplifier Research), was aligned epi/endocardially with its focus placed slightly (<0.7 mm) beneath the transmural surface of the ventricle. The HIFU transducer (focal length 56.5 mm and 6-dB lateral width 0.6 mm and axial width 7 mm) was calibrated using a custom fiber optic probe hydrophone [25]. Optical mapping used two green-filtered light emitting diodes (531±45 nm, 5G Illumination, SciMedia USA) for excitation and a 100×100 pixel CMOS camera system (MiCAM Ultima-L, SciMedia USA) to record the long-pass filtered (>650 nm) emission fluorescence from the wedge transmural surface (1000 frames/s, spatial resolution 200 – 260 µm/pixel). Two dimensional M-mode (M2D) ultrasound imaging (11 frames/s) was performed using a Vevo 770 system (VisualSonics, Inc.) with a RMV 707B probe (30 MHz, 12.7 mm focal length, 2.2 mm 6-dB focal depth, 45 MHz bandwidth) which was orthogonally aligned with the CMOS camera to image the same tissue transmural plane (Figure S1). A triangular cut on the edge of the polycarbonate screen (Figure 1B) served as a fiducial marker for image registration.

A custom-programmed FPGA board (Cyclone® II, Altera) was used to synchronize optical mapping (0 to 32 s), M2D ultrasound imaging (4 to 8.6 s), and HIFU ablation (4.5 to 8.5 s). HIFU pulses were interleaved with ultrasound imaging to avoid interference. Backscattered radiofrequency (RF) signals of ultrasound imaging were digitized using an 8-bit oscilloscope (54830B, Agilent Technologies) at 100 Msample/s for off-line analysis. Pulsed HIFU (pulse-repetition-frequency 11 Hz, duty cycle 70%, duration 4 s, spatial-peak pulse-average intensity 3500 W/cm^2^) was applied to generate a single lesion near the wedge cross-sectional (transmural) surface to allow spatiotemporally correlated optical mapping and ultrasound imaging.

### Optical Mapping Data Analysis

Optical mapping images were analyzed as described previously [26]. Briefly, 3×3 spatial binning and 1 – 100 Hz FIR band-pass filter were applied for spatiotemporal smoothing. Photobleaching related and HIFU induced baseline drift of optical recordings was corrected using linear, logarithmic, and polynomial fitting. The measured optical action potentials (OAPs) were then normalized to be between 0 and 1. Activation time was determined as the time needed for d(ΔF)/dt to reach maximum during each cardiac cycle, where ΔF is the fractional fluorescence change (change of fluorescence signal divided by background fluorescence). Activation delays between adjacent pixels were calculated using spatial gradient method and divided by the distances between pixels to calculate conduction velocity (CV) vectors. AP duration at 50% and 80% repolarization (APD_50_ and APD_80_) were calculated as the duration from the activation time to 50% or 80% of the peak AP amplitude (APA) during repolarization. Degree of AP triangulation was quantitatively characterized by the changes of the ratio between APD_50_ and APD_80_, and AP upstroke rising rate was defined as [d(ΔF)/dt]_max_. Changes of each AP parameters are calculated by subtracting pre-HIFU frames (1 – 4 s, Figure S2).

### Parametric Ultrasound Imaging

Amplitudes of the Hilbert transformed RF signals of ultrasound images, or envelope data *A*(*x*, *y*; *t*) (*x* and *y* are the 2D locations, and *t* is a time index), were used to form various parametric images as described previously [18], [27], [28]. Briefly, an image parameter, *X*(*x*, *y*; *t*) (e.g. Grayscale, IBS, Rayleigh and lognormal parameter, etc.), was computed for each pixel to form a parametric image. It is color-coded to overlay on the original unprocessed image (Figure S3). Gray scale (GS) and IBS images were computed as [18]



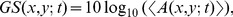
(1)

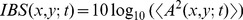
(2)


respectively. Historically, first-order statistical models have been fitted to ultrasound backscattered signals for tissue characterization, and among all, Rayleigh distribution was demonstrated a suitable model for characterizing echoes from myocardium [16], [27], [29]. In this study, Rayleigh parameter (*α*) and log-normal scale parameter (σ), reflecting spatial distribution of ultrasonic scatters, were calculated based on the spatial intensity distribution of the envelope data using maximum likelihood estimates [27], [28]



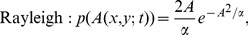
(3)




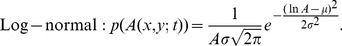
(4)


The following parameters were computed to illustrate temporal evolution for a given parameter 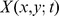




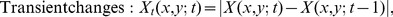
(5)




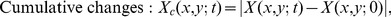
(6)




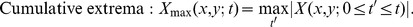
(7)where 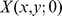
represents the signal averaging of pre-HIFU frames (1 – 4 s).

### Histology and Lesion Assessment

Before and after each experiment, the transmural surface of the wedge was photographed, and images were taken again after wedges were stained with triphenyltetrazolium (TTC, Sigma Aldrich) [30]. Wedges after each experiment were stored in 10% formalin for >48 hours, sectioned with a 200 µm step-size from the imaging plane of the wedge preparation. Formalin-fixed tissue blocks were sectioned at 5 µm thickness for masson's trichrome (MT) staining and stained slides were scanned using a high resolution scanner (CanoScan 8800F, Canon). HIFU induced lesions were identified using a Matlab algorithm. Briefly, RGB images of MT slides were converted into L*a*b* color space (Matlab function: *makecform* and *applycform*) and classified into two groups using k-means clustering method. The image segments corresponding to HIFU lesions were picked out and diluted using a Markov random field model. The resultant lesion masks were then compared with manually segmented lesions from TTC stained images.

### Image Registration

**Figure 2:**
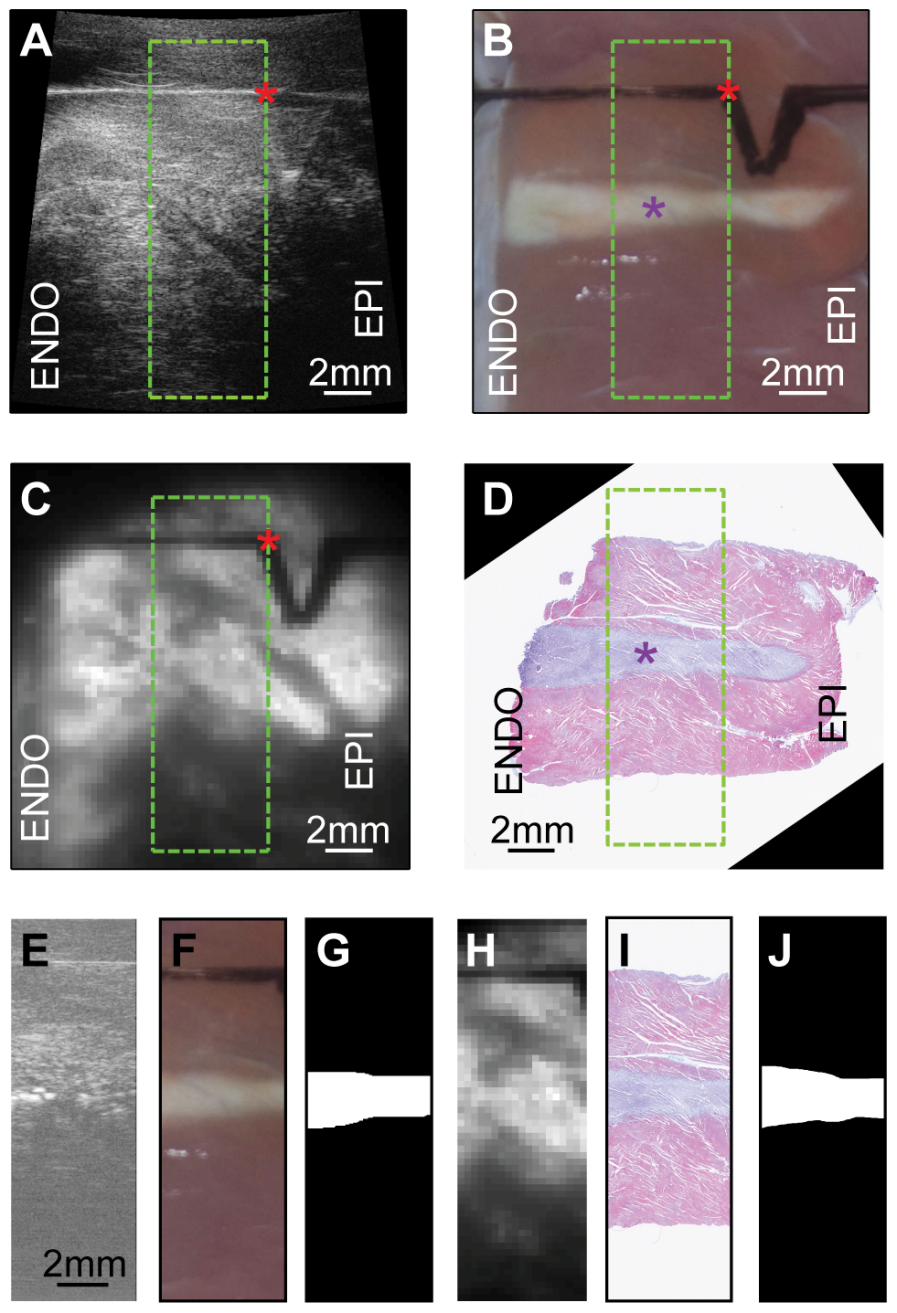
Demonstration of image registration. (A) Ultrasound B-mode image of a wedge preparation acquired after HIFU ablation with endocardium (ENDO) on the left and epicardium on the right (EPI). Sharp corners (red asterisk) of a “V” shape carving marker on the acoustic transparent screen show hyperechoic signals, making it a suitable fiducial marker for image registration. Green box indicates the field-of-view (FOV) of M2D mode imaging. (B) Photograph of a canine wedge preparation after HIFU ablation. Purple asterisk labeled the lesion centroid. (C) Background optical mapping image of the same wedge preparation. M2D mode image, gross image, and optical images were registered based on the FOV and fiducial marker. (D) Masson's trichrome (MT) stained microscopic slide of the same transmural plane. E – J, Registered FOV of M2D-mode image, gross photograph, lesion mask from gross photograph, background optical image, MT slide, and lesion mask from MT image.

Ultrasound images, gross tissue photographs, and optical mapping images were registered using the fiducial marker carved on the flat acoustic transparent screen (Figure 2A – C). MT images and gross photographs were registered based on lesion geometry. An example of registered images was shown in Figure 2E – J.

### Statistical Analysis

Receiver operating characteristic (ROC) analysis was performed to determine whether HIFU induced ΔAPA corresponded to lesion and to test ultrasound imaging parameters for detecting lesion and ΔAPA. Leave-one-out (LOO) cross-validation was employed to determine the variation of ROC analysis. Results are presented as mean ± standard deviation. Kruskal-Wallis ANOVA was used for comparing groups and *p*<0.05 was considered statistically significant.

## Results

### Transmural Optical Mapping of Perfused Canine Cardiac Wedge Preparations

**Figure 3:**
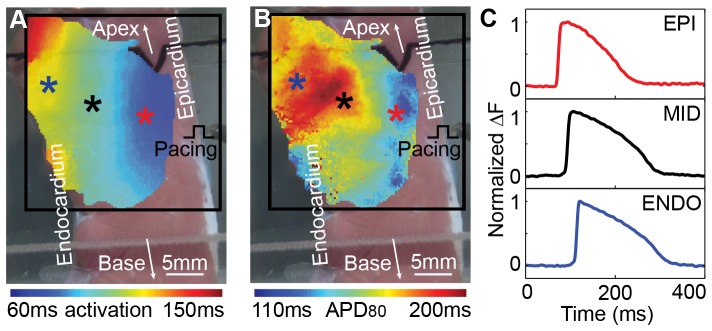
Electrophysiology and optical action potentials (OAPs) of a canine left ventricular wedge preparation. (A) Activation map superimposed on the photograph of the transmural surface of the wedge. Black box is the region of interest. The color asterisks indicate representative locations across ventricular wall. Pacing (cycle length = 1000 ms) was applied from the epicardium. (B) APD_80_ map superimposed on the same gross photograph of the transmural surface. (C) Representative OAPs at subepicardium (EPI), midmyocardium (MID), and subendocardium (ENDO) indicated by the colored asterisks in (A – B).

The OAPs, activation pattern, and APD measured from the perfused wedge preparations in this study, whether pacing endocardially (n = 5) or epicardially (n = 8), were consistent with reported results [23]. As an example, a canine LV wedge preparation under a pacing rate of 60 bpm (CL = 1000 ms) exhibited systematic activation (Figure 3A) and steep spatial APD_80_ gradient (Figure 3B) transmurally with shorter APD_80_ near the epicardium and longer APD_80_ near the endocardium. AP morphology (Figure 3C) at different layers was consistent with that under normal sinus rhythm, indicating sufficient perfusion, cell-cell coupling, and absence of ischemic regions in the preparations. The perfused preparations were viable with normal EP for over 3 hours during which all experiments were performed.

### Transmural EP Changes associated with Lesion generated by HIFU Ablation

**Figure 4:**
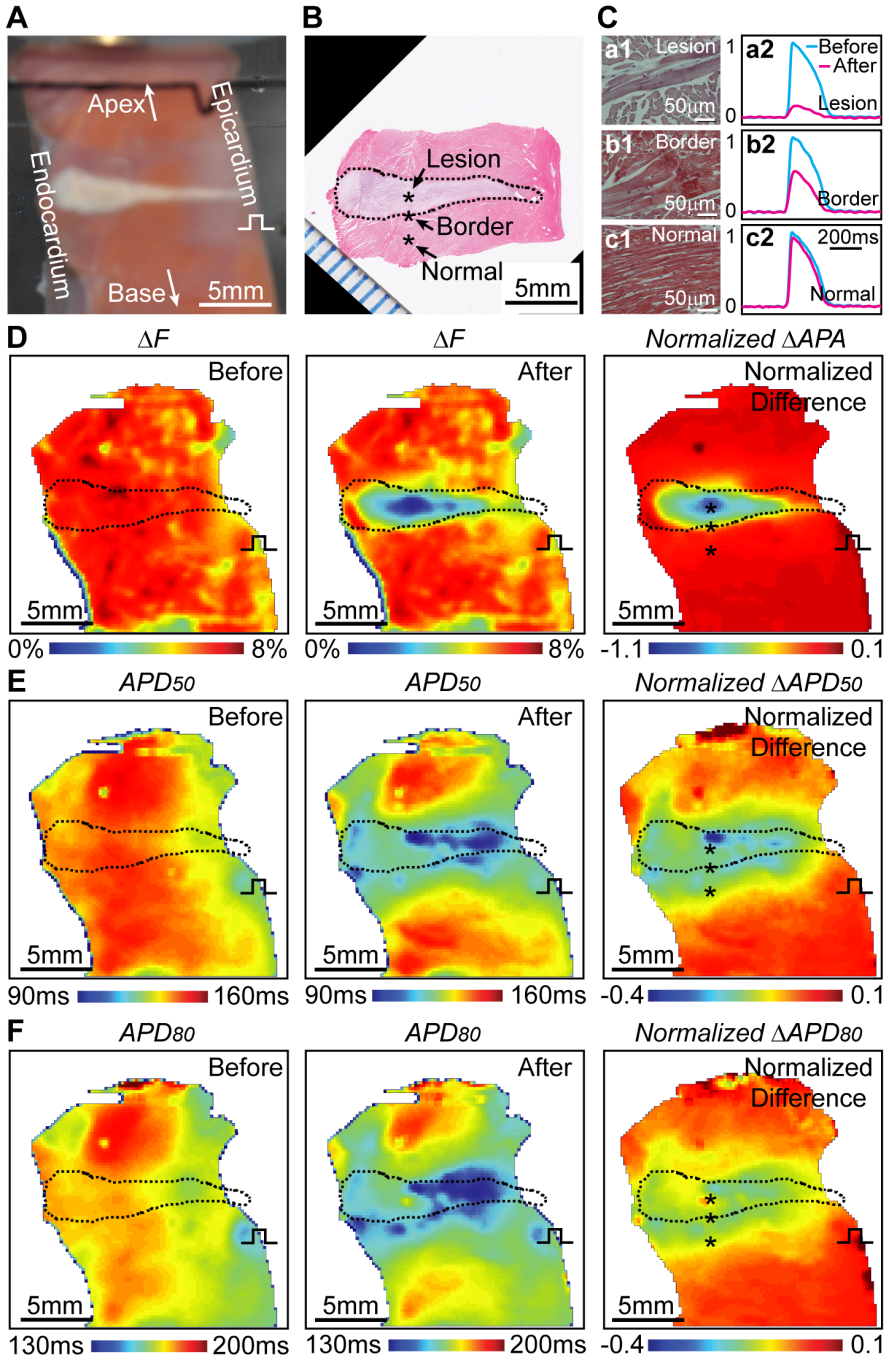
Spatiotemporal changes of transmural AP surrounding HIFU lesion. (A) Photograph of a left ventricle wedge with a HIFU lesion. (B) Masson's trichrome (MT) stained slide corresponding to the same tissue plane in A. Black dash line encircles lesion boundary. Black asterisks labeled representative locations at inside lesion, border, and normal tissue. (C) Microscopically magnified (40X) MT images at labeled locations in B: lesion (a1), border (b1), and normal tissue (c1). Corresponding normalized OAPs (a2, b2, c2) before (blue) and after (pink) HIFU ablation. (D) Amplitude of fractional fluorescence intensity (ΔF) before and 24 s after HIFU ablation, and change of action potential amplitude (ΔAPA) with dashed line highlighting the same lesion in B. (E) APD_50_ before and after HIFU ablation and ΔAPD_50_. (F) APD_80_ before and after HIFU ablation, and ΔAPD_80_.

HIFU generated single lesions, either cigar-shaped (n = 4) or tadpole-shaped (n = 9), as indicated by the pale or discolored region in the gross photograph (Figure 4A) and MT stained slide (Figure 4B), were (12.27±2.03) mm×(2.86±0.57) mm (n = 13) along the depth of ventricular wedges. Lesion regions detected from MT images, TTC stained images, and gross photographs showed high similarity (>98% pixel-by-pixel match, Figure S4).

Microscopically, condensed cytoplasmic filament and loss of cellular structure were observed near the central necrotic area (Figure 4C-a1). A border zone was seen as a rim of necrotic contraction band with apparent hemorrhage or inflammation but intact cell structures (Figure 4C-b1) with thicknesses of 327.6±69.3 µm (n = 13). In the normal tissue far away from the border zone, cardiomyocytes were intact (Figure 4C-c1). Nine out of the thirteen lesions showed small microscopic cavities on the lesion MT slides with the lesion shapes being tadpole-like and cavity areas being 0.30±0.25 mm^2^, suggesting transient effects from high temperature related “boiling” or HIFU induced cavitation. Correspondingly, different EP characteristics were observed within and surrounding HIFU lesions in the ventricular transmural plane. Near the lesion center, significant APA reduction (∼80%) was accompanied by significantly more APD shortening, AP triangulation, and reduced upstroke rising rate. Fewer changes were seen in the border zone (Figure 4C-b2) and no changes were observed in tissue regions further away (Figure 4C-c2).

**Figure 5:**
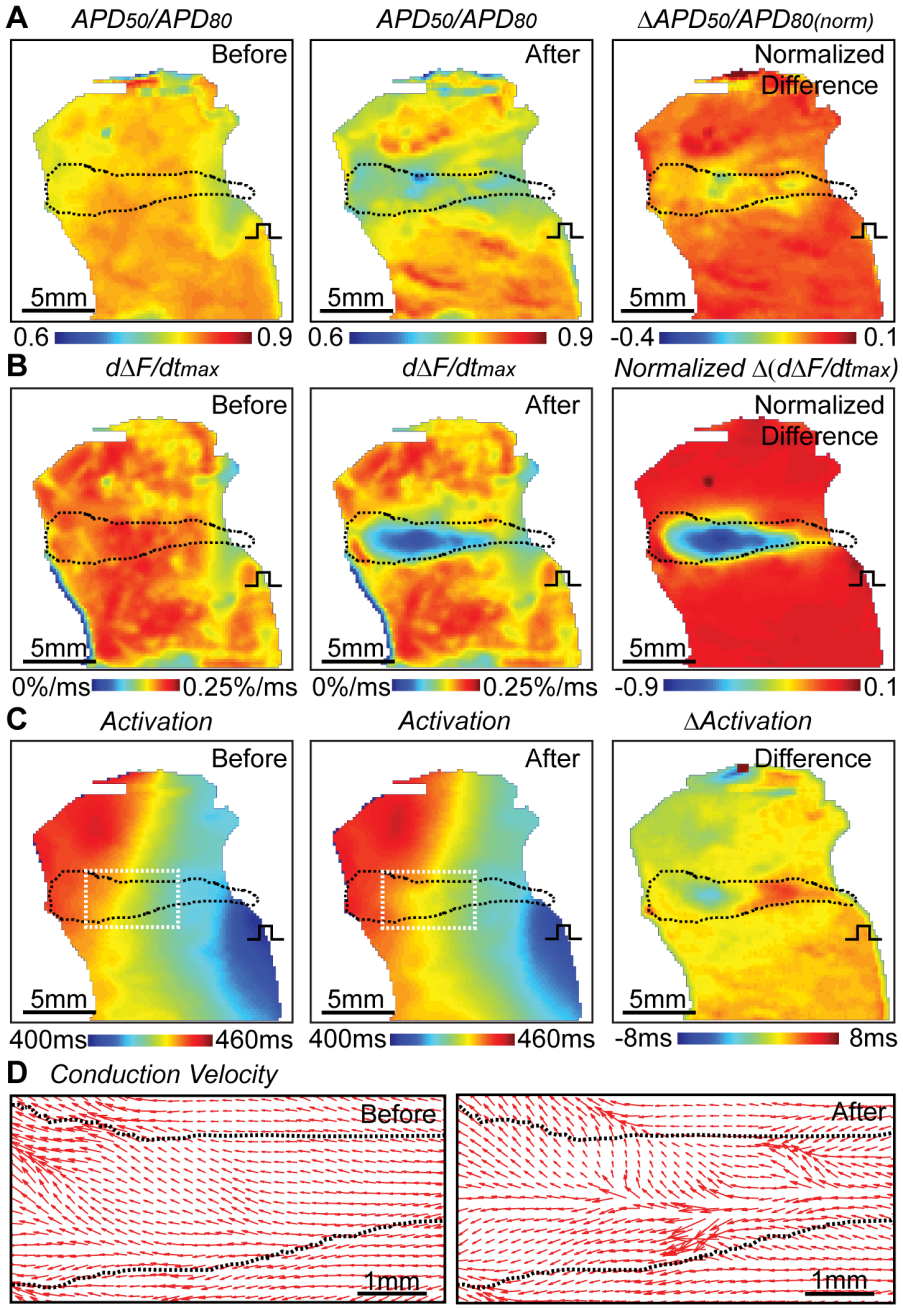
Spatiotemporal AP triangulation, excitability, and activation pattern for HIFU ablation. (A) Ratio of APD_50_ and APD_80_ before and after HIFU ablation, and ΔAPD_50_/APD_80_. (B) Upstroke rising rate (dΔF/dt)_max_ before and after HIFU, and their difference. (C) Activation time before and after HIFU, and their difference. (D) Conduction velocities (CVs) before and after HIFU ablation in the white boxed ROI in the activation maps in (C). Directions of electrical wave propagation and magnitude of CVs are indicated by the directions of the red arrows and their lengths. Same lesion area as in Figure 4 is indicated by black dash lines.

Overall higher APA reduction was seen near lesion center than in border zone (Figure 4D) (n = 13). Similarly, APD_50_ and APD_80_ shortening (up to 33% and 21% respectively) were much severer near the center than in border zone. However, shortening of APD extended beyond lesion boundary or region of APA reduction, up to 2.8 mm for APD_50_ (Figure 4E) and 2.0 mm for APD_80_ (Figure 4F). Transmurally, more APD reduction was seen near epicardium than endocardium, implying different thermal sensitivity of APD across ventricular wall. In the total thirteen HIFU ablations, nine wedges showed more APD reduction at epicardium than endocardium regardless of the orientations of the wedge preparations from the HIFU transducer. Decrease of APD_50_/APD_80_ (from 0.8 to 0.74) suggested influence of HIFU on the plateau potential (Figure 5A). Suppression of cellular excitability within lesion after HIFU ablation was indicated by a reduction of upstroke rising rate from 0.19%/ms to 0.11%/ms (Figure 5B). Although no transmural conduction block for the single lesion on the depicted LV wedge was seen on activation map, local activation delay up to 5 ms was observed closer to the epicardial pacing site and minor early activation (2 ms) occurred in the lesion center (Figure 5C). The conduction wavefront bifurcated in the lesion axial direction and increased CVs were seen along the edge of the lesion (Figure 5D). Bipolar pacing confirmed loss of excitability in lesions (Figure S5). No propagating APs were observed when pacing at lesion center with 2X or 3X of the threshold voltage (3 V), while pacing provoked AP propagation was generated when pacing was near the lesion border and normal tissue.

### Spatiotemporal Characteristics of AP Changes during HIFU Ablation

**Figure 6:**
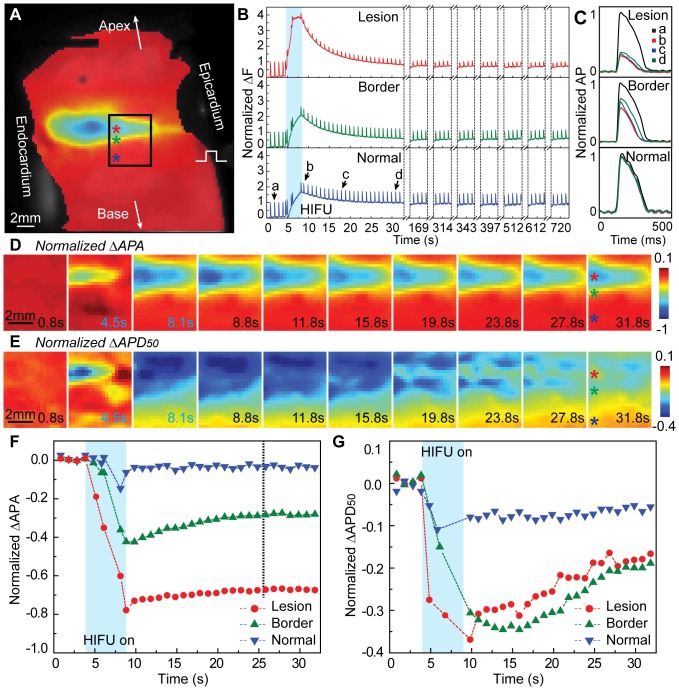
Acute changes of AP prior, during, and after HIFU ablation. (A) Changes of OAPs amplitude (ΔAPA) at *t* = 32 s (colored) superimposed on background optical mapping image (gray scale). Black box represents the ROI (6mm×5.6 mm) within the field-of-view of M2D ultrasound imaging. Colored asterisks labeled representative locations inside lesion, border, and normal tissue. (B) Normalized OAPs at the representing locations in (A) with each “spikes” corresponding to single cardiac cycle. Cyan shaded area indicates HIFU duration. Single cycle OAPs at *t* = 1.8, 8.8, 17.8, and 31.8 s indicated by a, b, c, and d. (C) Normalized OAPs at the representative locations in (A) superimposed at representative times (arrows a – d in B). (D) Spatiotemporal changes of ΔAPA. Blue labeled frames were during HIFU ablation. (E) Spatiotemporal changes of ΔAPD_50_. (F) Normalized ΔAPA at representative locations (asterisks in D and E). (G) Normalized ΔAPD_50_ at the same representative locations.

Baseline change of fluorescence signals (ΔF) from optical mapping was observed during HIFU ablation (Figure 6B). A separate unpublished data suggested that such baseline changes correlated with tissue temperature changes induced by HIFU ablation. Analysis of EP changes in this study was performed after baseline drift correction.

In addition to spatial distribution of OAP changes (Figures 6A, 6C and Figure 4C), we observed that OAP exhibited little recovery within lesion but partial recovery in the lesion border zone after HIFU application (Figure 6C). Compared to HIFU induced APA reduction (Figures 6D, 6F), APD_50_ shortening extended to larger spatial region (Figures 6E) and occurred faster than ΔAPA during HIFU application (shaded areas in Figures 6F and 6G). In addition, less recovery of APA than APD_50_ occurred after HIFU application when tissue gradually cooled down. These observations indicate that plateau potential (related to APD) is more thermally sensitive than excitability (associated with APA and resting membrane potential).

### HIFU Lesion vs. OAP Changes

**Figure 7:**
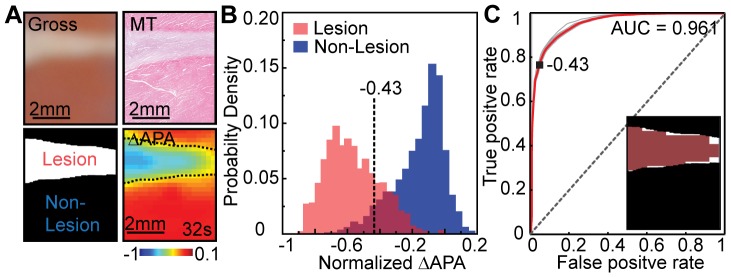
OAPs changes within and outside a HIFU lesion. (A) Photograph and masson's trichrome (MT) stained slide of a wedge preparation after HIFU ablation (top panels) along with the corresponding binary lesion mask (lesion as white and non-lesion as black) (bottom left panel), ΔAPA obtained at *t* = 32 s. Black dash lines highlight lesion edges. (B) ΔAPA (n = 13) within lesion and outside lesion (non-lesion) determined by lesion mask. A black dash line indicates a threshold (−0.43±0.01) of ΔAPA used to predict irreversible lesions determined from receiver-operating characteristic (ROC) analysis. (C) ROC curves for ΔAPA detection of lesion for all cases (individual and overall curve are marked gray and red respectively) with an area under ROC curve (AUC) of 0.961. Inset: ΔAPA predicted lesion (red) for the representative example in (A) is overlaid on the real binary lesion mask.

We determined the threshold of ΔAPA corresponding to HIFU lesion. Based on a lesion mask obtained from gross photograph and MT slide with corresponding ΔAPA map at *t* = 32 s (Figure 7A), histograms of the ΔAPA values at pixel locations inside (2058 pixels) or outside lesion mask (4117 pixels) (n = 13) (Figure 7B) show that APA reductions inside lesion (−0.57±0.16) was significantly higher than outside (−0.16±0.15) (*p*<<0.05). The ROC curves (Figure 7C) of ΔAPA using all preparations yielded an averaged ROC area-under-curve (AUC) of 0.96±0.01. The optimal threshold of ΔAPA for detecting lesion was −0.43±0.01. LOO test yielded an ROC AUC of 0.88±0.07, confirming stability of the detection method.

### Detection of Lesion using Optical Mapping and Ultrasound Imaging

**Figure 8:**
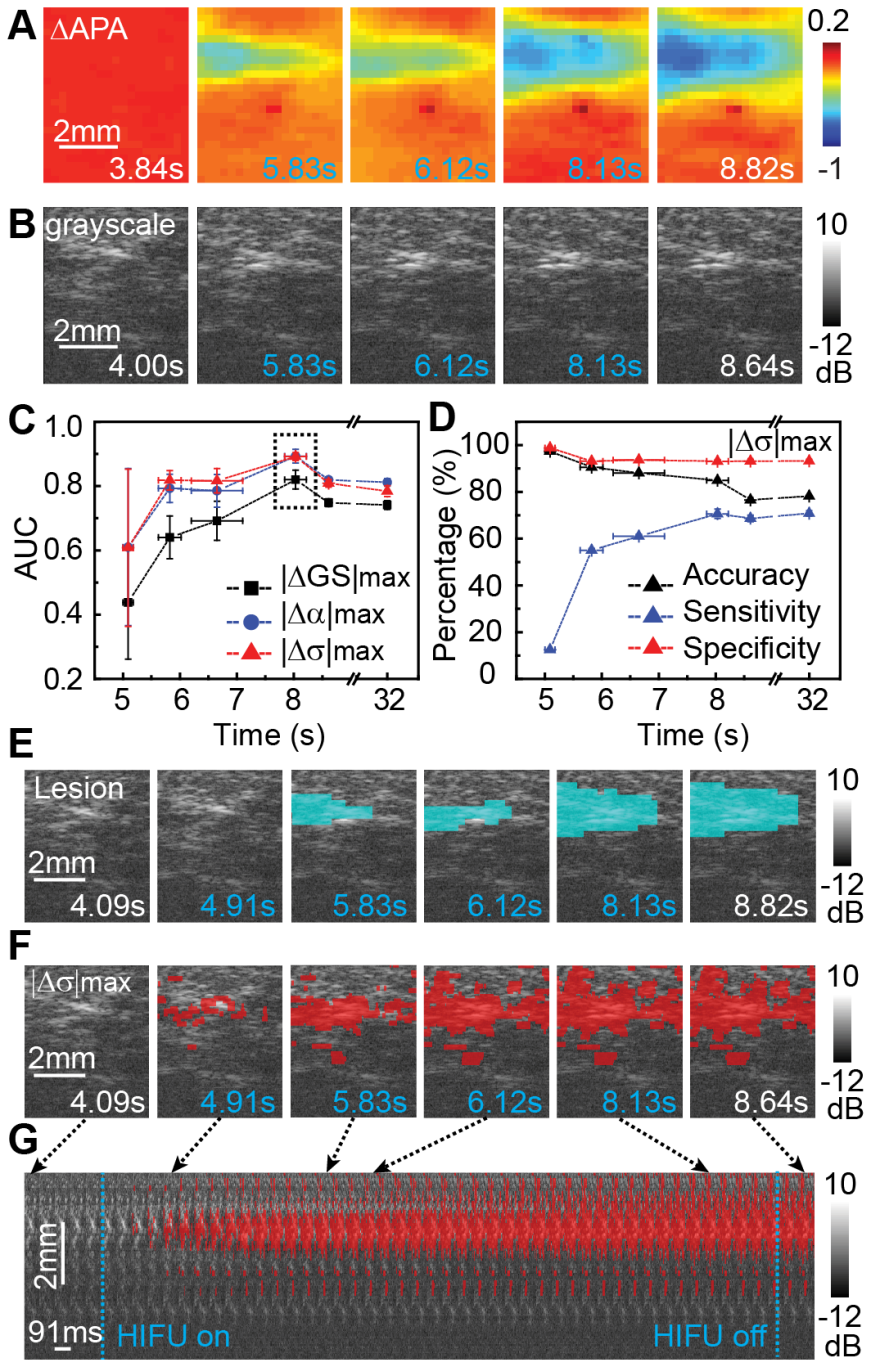
Lesion detection using parametric ultrasound imaging and optical mapping. (A) Selected ΔAPA maps prior, during, and after HIFU ablation. HIFU ablation was applied from *t* = 4.55 to 8.55 s. (B) Selected grayscale ultrasound images (from *t* = 4 to 8.64 s) corresponding to the ΔAPA maps in (A). Frames during HIFU ablation are labeled with blue time indices. (C) Temporal change of the ROC area-under-curves (AUCs) for |ΔGS|_max_ (black), |Δ*α*|_max_ (blue), and |Δσ|_max_ (red), with maximum at *t* = 8.03±0.19 s (black dash box). (D) Temporal changes of detection accuracy, sensitivity (true positive rate), and specificity (true negative rate) using |Δσ|_max_. (E) M2D-mode images color-coded by lesion map determined by ΔAPA threshold (cyan). (F) M2D images color-coded by lesion region determined by cumulative extrema of log-normal parameter |Δσ|_max_ (red). (G) Stacked temporal M2D images superimposed with lesion mask detected by |Δσ|_max_.

A clear region of APA reduction was readily identified from optical mapping (Figure 8A), but lesion identification was difficult from regular B-mode gray-scale ultrasound images (Figure 8B).

Assuming ΔAPA threshold of −0.43±0.01 corresponded to lesion, parametric images using |ΔGS|_max_, |Δ*α*|_max_, and |Δσ|_max_ yielded the best AUCs, 0.82±0.03, 0.89±0.02, and 0.89±0.01 respectively (Table1), much better than conventional B-mode (0.53±0.01). The values of ROC AUCs indicate a good correlation between lesion size predicted with the ultrasound parameters and histological lesions. The ROC AUCs increased significantly until the end of HIFU application (*t* = 8 s, n = 4) (Figure 8C), as detection sensitivity increased correspondingly (Figure 8D). Since |Δσ|_max_ generated the highest ROC AUCs (optimal threshold 0.28±0.01), we chose it for our subsequent analysis.

**Table 1:** The area under the receiver-operating characteristic curve (ROC AUC) for detection of lesion from various ultrasound parameters on all canine wedges (n = 13).

Parameter	Symbol	ROC AUC	Threshold
Gray-scale change (tran.) [dB]	|ΔGS|*_t_*	0.53±0.01	5.09±1.84
Gray-scale change (cumu.) [dB]	|ΔGS|*_c_*	0.64±0.03	5.43±1.28
Gray-scale change (cumu. extrm.)	|ΔGS|*_max_*	0.82±0.03	0.44±0.16
IBS change (cumu.) [dB]	|Δ*IBS*|*_c_*	0.65±0.03	10.50±2.10
IBS change (cumu. extrm.)	|Δ*IBS*|*_max_*	0.71±0.06	0.46±0.22
Rayleigh *α* change (tran.)	|Δ*α*|*_t_*	0.69±0.04	0.11±0.14
Rayleigh *α* change (cumu.)	|Δ*α*|*_c_*	0.77±0.03	0.16±0.03
Rayleigh *α* change (cumu. extrm.)	|Δ*α*|*_max_*	0.89±0.02	0.11±0.02
Log-normal σ change (tran.)	|Δσ|*_t_*	0.64±0.05	0.12±0.11
Log-normal σ change (cumu.)	|Δσ|*_c_*	0.75±0.01	0.18±0.01
Log-normal σ change (cumu. extrm.)	|Δσ|*_max_*	0.89±0.01	0.28±0.01

“tran.”, “cumu.”, and “cumu. extrm.”. transient, cumulative, and cumulative extrema of each ultrasound parameter changes are indicated as

Figures 8E – 8G show an example of color-coded parametric ΔAPA (blue) and |Δσ|_max_ (red) images showing temporal lesion evolution. Compared to ΔAPA detected lesion (Figure 8E), |Δσ|_max_ imaging overestimated lesion area at the beginning of HIFU ablation, but improved significantly and achieved good lesion prediction in the end.

### Parametric Ultrasound Imaging vs. ΔAPA

**Figure 9:**
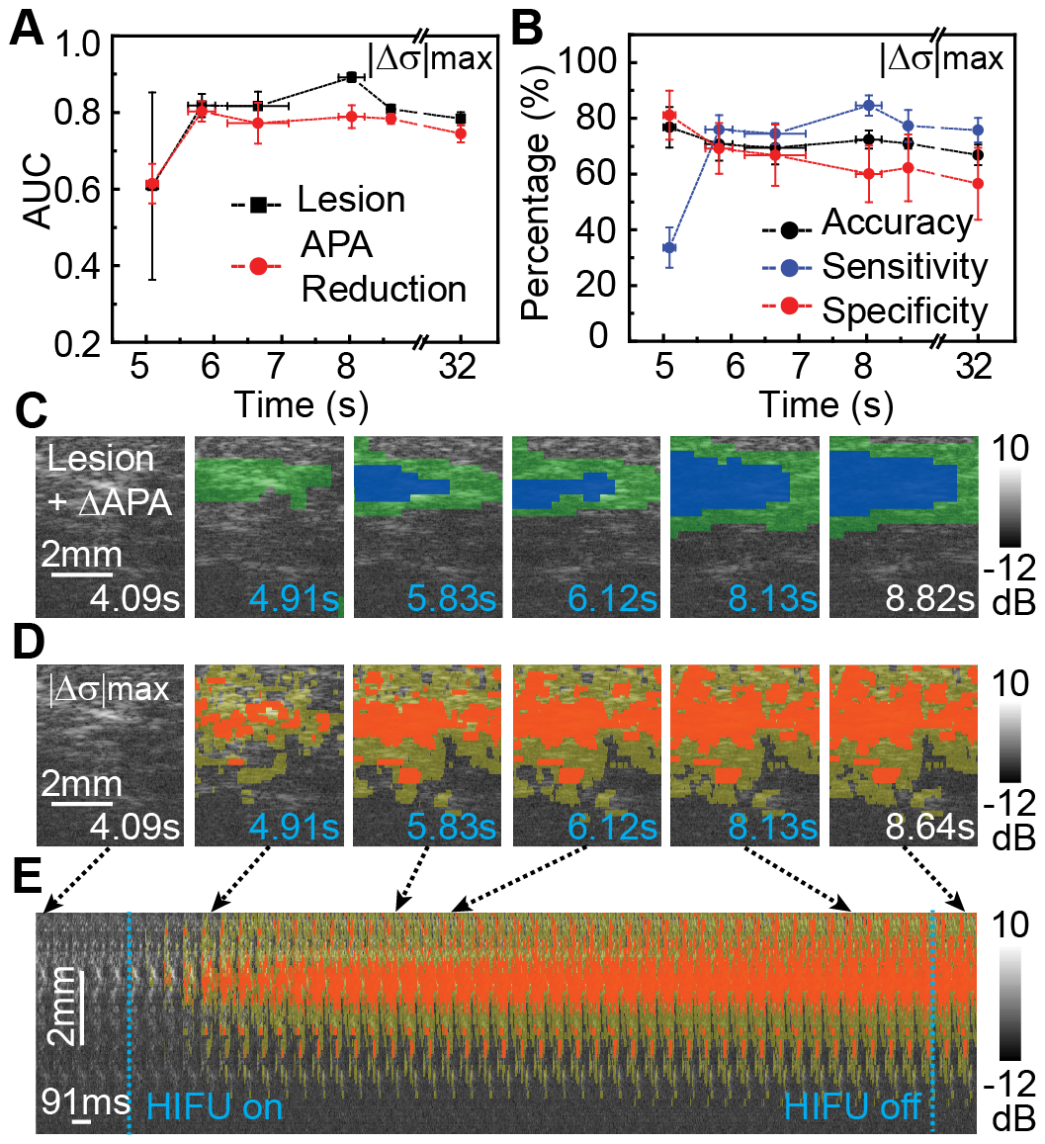
Spatiotemporal correlation of APA with parametric ultrasound imaging. (A) Temporal changes of the ROC area-under-curves (AUCs) using |Δσ|_max_ for detecting lesion and ΔAPA below lesion threshold (non-lesion). (B) Temporal changes of detection accuracy, sensitivity (true positive rate), and specificity (true negative rate) using cumulative extrema of log-normal parameter (|Δσ|_max_) for detection of lesion and peripheral ΔAPA regions. (C) M2D images color coded by true lesion region (ΔAPA<–0.43, blue) and non-lesion ΔAPA (–0.43<ΔAPA<–0.19, green) determined from ΔAPA maps. (D) Gray-scale M2D images color coded with lesion (red) and non-lesion ΔAPA (yellow) regions determined from |Δσ|_max_ images. Blue time indices indicate frames during HIFU application. (E) Stacked temporal M2D images coded with lesion and non-lesion ΔAPA masks detected by |Δσ|_max_.

In addition to detecting ΔAPA associated with lesion (<−0.43), distribution parameters *α* and σ outperformed other parameters in detecting ΔAPA regions (between −0.19 and −0.43) that were in vicinity of the lesions during HIFU ablation. An arbitrary value −0.19, significantly above the noise level (−0.07±0.12), was chosen as the detectable ΔAPA regions. Again, the cumulative extrema of the parameters yielded higher AUC than transient and cumulative changes. Figure 9A shows the ROC AUCs of |Δσ|_max_ for detecting ΔAPA associated with lesion and non-lesion at different times. Compared with ROC AUC of |Δσ|_max_ for detecting lesion-related ΔAPA (0.89±0.01), non-lesion ΔAPA detection achieved an AUC of 0.79±0.03. Similarly, ROC AUCs improved with time, with an increase of detection sensitivity correspondingly (Figure 9B).

Figure 9C shows an example of color coded ΔAPA images for lesion (ΔAPA<−0.43) in blue and non-lesion regions (−0.19<ΔAPA<−0.43) in green. Figure 9D is the color coded |Δσ|_max_ images for detecting lesion ΔAPA (red) and non-lesion (yellow), with an overestimation of the area associated with non-lesion ΔAPA.

## Discussion

Using optical mapping and ultrasound imaging, we investigated HIFU induced tissue and EP changes transmurally in a spatiotemporally correlated fashion in perfused canine wedge preparations. We demonstrated that parametric ultrasound imaging using cumulative extrema of the log-normal scale parameter σ (|Δσ|*_max_*) can image HIFU generated lesion and ΔAPA around the thermal lesions. To note, the proposed imaging technique, which is potentially useful for intra-operative identification of lesion transmurality, can be applied in RF and laser ablation as well, and help improve the efficacy of current ablation procedure in clinical practice.

### Spatiotemporal Changes of APA and APD surrounding HIFU Lesions

Our results of the spatiotemporal changes of EP induced by HIFU ablation, such as reduction of APA and shortening of APD (Figure 4 and Figure 5), in the transmural surface of the wedge preparations are in general agreement with reported EP data recorded epicardially and intracardially [21], [31], [32]. HIFU generated lesion and EP changes in our study showed similar properties to RF lesions. However, several findings are worth noting.

Significant APA reduction (e.g. 80%) has been used clinically to confirm ablation [33] but no universal endpoint for single ablation has been well defined. In this study, we determined a threshold of ΔAPA (43±1%, n = 13) that best demarked single lesion region validated by histology (Figure 7). The more conservative reduction may be necessary clinically to reduce the risk of electrical-reconnection.

Shortening of APD_50_ and APD_80_ extended beyond the lesion boundary (Figures 4 and 6), as well as triangulation (Figure 5), consistent with reported results using RF ablation [21]. APD_50_ and APD_80_ occurred faster than and prior to APA reduction (Figures 4 and 6), and there was also larger recovery of APD than APA after HIFU application with tissue cooling, suggesting a more thermally sensitive characteristics of the plateau potential ion channels than cellular excitability and a more stable measurement of EP changes using APA instead of APD. Both APD_50_ and APD_80_ outside the lesion fully recovered 218.8±96.6 s after HIFU ablation (n = 12). In addition, less reduction in APD_80_ compared to APD_50_, firstly reported by Wood *et al.* in RF ablation [21], was also observed within HIFU lesion. Triangulation was <20% within and in lesion border zone (Figure 5). The dominant mechanism was speculated to be the disproportional inactivation or injuries between L-type Ca^2+^ channels and delayed outward K^+^ channels when HIFU induced temperature across certain threshold.

Optical mapping in a transmural surface revealed early activation within the HIFU lesion and local activation delay at locations proximal to pacing site, suggesting the effect of the intricate alignment of myocardial fibers across the transmural ventricular wall (Figure 5). CVs increased along the edges of HIFU lesions, similar to the observations in RF lesions [21], [34]_ENREF_29. However, due to the smaller lesion size and photon scattering, the recorded activation and conduction velocity within the lesion can also be scattered optical signals from the viable tissue surrounding the lesion [35]. Therefore, cautions are needed when interpreting the optical mapping data at the lesion area.

### Lesion Assessment using Ultrasound Imaging

Superior tissue penetration of ultrasound imaging makes it a suitable tool for real time monitoring of lesion formation along tissue depth, as observed in RF ablation in an *in vivo* sheep model [15]. However, conventional ultrasound imaging has not provided sufficient contrast to detect lesions accurately.

Eyerly *et al.* demonstrated an novel ICE based ARFI imaging technique using a 6.15 MHz imaging probe for identifying RF generated lesions and discontinuous lesion set [19]. But the imaging rate of ARFI was <4 frames/min and image quality was affected by the imaging probe placement and cardiac motions. Wright *et al.* introduced a novel system that combined ultrasound imaging with ring or TPX-covered RF catheters to directly visualize lesion formation in real time [15]. We have previously shown that high frequency imaging at 55 MHz combined with fast frame rate M-mode (1 kHz) and short time B-mode (STBM) (frame rate 70 – 130 Hz) can image HIFU lesion and gas body formation [17], [18] by tracking the temporal history of changes in ultrasound integrated backscatter (*IBS*) and frame-to-frame echo decorrelation. In the current study, we employed ultrasound imaging at 30 MHz with imaging depth (∼12.7 mm) with better imaging depth more suitable for imaging across the depth of human atrial tissue (5 – 7 mm). Using the ΔAPA threshold corresponding to histological lesion, we showed that statistical parameters such as log-normal and Rayleigh parameters performed better than the parameters employed previously in predicting lesion formation and sizes.

Both Rayleigh and Non-Rayleigh distribution, such as K-distribution and Nakagami distribution, have been shown to differentiate infarcted myocardium from normal tissue with imaging frequency less than 15 MHz [27], [29]. In the current study, we showed that statistical parameters such as log-normal and Rayleigh parameters performed better than other parameters, suggesting the ultrasound scatters (e.g. cell nucleus) within HIFU lesions were most likely poly-dispersed and non-periodic [28]. The detection accuracies between lognormal and other non-Rayleigh distribution models (e.g. Nakagami and K distribution) were marginal. The phenotype of the distribution changed slightly after lesion formation and brought in large changes in the echogenicity which shifted the backscattered intensity to the higher end on the histogram, leading to a slight drop of goodness-of-fit using both lognormal and Rayleigh fitting (e.g. the adjusted R^2^ dropped from 0.99 to 0.98, and chi-squared values increased from 55.4 to 66.4 with the degree of freedom at 40 – 50 for lognormal fitting). In this study, nine ablations created tadpole-shaped lesion with microscopic cavity formation, indicating transient bubble activities. However, the current algorithm counted the microscopic cavity as a part of the lesion. The cumulative extrema of the parameters, which captured the temporal evolution of changes during HIFU ablation, performed better than transient and cumulative changes. For example, ROC AUC increased 20% using cumulative extrema of |Δσ|. To better assess the bubble activities, a frame-to-frame echo decorrelation method should be used where bubble expansion rate was calculated to predict the sizes of macroscopic cavities [18]. In addition, the spatial resolution with log-normal imaging was fine enough to image small size lesions (98 µm in axial and 115 µm in lateral), although slightly lower than the resolution of using grayscale and IBS (55×115 µm).

Compare to ARFI imaging which showed high sensitivity and specificity in detecting RF lesions, the current method achieved similar detection accuracy of HIFU lesions with higher frame rate and imaging resolution, thereby can capture rapid activities during ablation and provide a potential feedback control scheme. Furthermore, our imaging method is less restricted by the probe placement angle, and does not depend on the quality of acoustic radiation force which can be challenging for imaging tissue at depth using ARFI. Unlike ARFI imaging which needs specific conditions only available after experimentation [36], our detection algorithm can detect lesion automatically using simple clustering method (e.g. k-means clustering) despite that we employed a more rigorous ROC training process. However, multiple lesions or lesion lines cannot be imaged simultaneously using our current method since it requires the time history of ultrasound backscattered signals for each individual lesion whereas ARFI imaging can be more advantageous in such case. As our ultrasound imaging technique is a point-by-point based detection of lesion transmurality, a potential translation to clinical practice is through combination with ablation catheters. By registering ultrasound detected transmural lesion with electroanatomical maps, multiple lesions can be planned side-by-side to form complete circumferential or linear lesion in pulmonary vein isolation procedure.

### Distinguishing Irreversible and Reversible AP Changes by Parametric Ultrasound Imaging

Our results show that histological lesion region matched well with spatial map of APA reduction above a threshold (Figure 7), suggesting a link of cell morphological damage (e.g. loss of cellular nuclei, myocardial fiber dissolution, damaged plasma membrane and gap junctions) (Figure 4C) with cellular EP change including the loss of AP and inter-cellular electrical conductivity [37].

Although it is the irreversible ΔAPA correlated to lesion that is relevant for intra-operative ablation guidance, it is important to gauge smaller changes of APA, as Wood *et al.* observed that the EP within lesion border zone completely recovered 22±13 days after RF ablation [21], which may be responsible for arrhythmia recurrence. We demonstrated that parametric ultrasound imaging can accurately detect lesion thus irreversible changes of APA during HIFU ablation and also smaller APA changes although with lower accuracy.

The spatiotemporal correlation between the lognormal parametric images and both lesion and peripheral APA changes appeared to be weak at early stage of HIFU ablation (Figure 8 and 9). Several reasons can help explain: (1) lesion detection based on APA maps may not be sufficiently sensitive to capture small volume tissue changes when lesion just began to form; (2) the lesion thresholds for both APA and ultrasound parameters were determined after HIFU application when lesion was mostly stabilized thereby may not be applicable at early stage of lesion formation; (3) transient activities (e.g. transient gas pocket formation) occurred at the beginning of HIFU ablation can cause false detection on ultrasound imaging; (4) lesion thresholds based on ROC analysis had variations while we only implemented the mean values for lesion mask calculation. Nevertheless, the compromised detection accuracy at the beginning of HIFU ablation did not affect the overall performance of lesion detection using ultrasound parametric imaging.

### Limitations

*In vitro* preparations of canine cardiac wedges that were mechanically stabilized using BDM have inherent physiological differences from *in vivo* beating hearts. A more appropriate decoupler, blebbistatin which has less side-effect on cellular EP than BDM [38], should be used. In this study ultrasound imaging and optical mapping were performed on the transmural tissue surface of a wedge preparation, which may be different from a transmural plane within a 3D tissue. Optical mapping only provided relative AP values and can only be used for *in vitro* studies. As we used a 30 MHz imaging system for high spatial imaging resolution, to extrapolate this to *in vivo* implementation for ablation monitoring, further studies are required to examine the effects of imaging frequency, imaging beam characteristics, as well as ECG-gating for motion tracking to suit for beating heart conditions. As a preclinical proof-of-principle study, we were able to image the lesion and AP changes on the ablation plane parallel to the ablation axis along tissue depth direction. Ideally, ultrasound imaging transducer shall be co-axially aligned with ablation probe as Wright *et al.* [15] presented earlier, therefore, further efforts are needed to fabricate dual-element transducers for co-axial ablation and imaging. Furthermore, the temperature during ablation was not monitored directly, thereby whether ablation induced “boiling” occurred and the effects of gas body activities and cavitation on the performance of the parametric imaging techniques for lesion and EP detection were not fully investigated.

## Conclusion

We revealed the spatiotemporal AP changes during HIFU ablation across the subepicardial tissue layers using perfused canine cardiac wedge preparations. Employing spatiotemporally correlated ultrasound imaging and optical mapping and validated by histology, we demonstrated that parametric ultrasound imaging using the cumulative extrema changes can detect thermal lesion that was correlated with irreversible APA changes along the tissue depth direction during HIFU ablation in real time.

## Supporting Information

Figure S1 **Relationship between optically mapped plane and ultrasound imaged plane.** (A) A canine left ventricular (LV) wedge preparation was mounted on a custom tissue holder with the transmural surface gently pushed against an acoustic transparent polycarbonate screen. Pacing was applied from epicardium (right). (B) The transmural surface of the LV wedge is optically mapped as demonstrated. Emitted fluorescence signals beneath the surface (<700 µm) were detected by CMOS camera for optical mapping. High frequency ultrasound imaging was performed from the top window of the tissue holder. Ultrasound imaging plane was adjusted through a high precision XYZ motor (0.05 µm step-size) to ensure the field-of-view (FOV) close enough to the LV wedge transmural surface. (C) A sequence of ultrasound B-mode imaging with 0.2 mm step size was performed to form a 3D ultrasound volumetric image to verify ultrasound imaged plane. Ultrasound B-mode image of the frontal plane of the transmural surface is presented. The top edge of flat imaging screen is visible in B-mode image. (D) Transverse plane of 3D ultrasound imaging volume with a solid red line highlighting the transmural surface of the LV wedge. (E) Sagittal plane of the same LV wedge with a red solid line representing wedge transmural surface and an orange thick line indicating the on-line ultrasound imaging plane during experimentation. Ultrasound imaged plane is verified <0.7 mm below the wedge transmural surface. (F) 3D comprehensive view of ultrasound imaged volume. A bright hyperechoic region close to the top is the edge of the flat screen.
(DOC)

Figure S2 **Example optical mapping data analysis.** (A) Sequential images (t_1_, t_2_, …, t_n_) were acquired via optical mapping on a canine left ventricular (LV) wedge. Temporal recording at each pixel corresponds to optical transmembrane action potential. Raw optical signal is filtered through a 1 – 100 Hz band-pass FIR filter and fractional fluorescence changes (ΔF) is used to describe optical action potentials (OAPs). (B) Within one cardiac cycle, map of ΔF amplitude before high-intensity focused ultrasound (HIFU) ablation is generated. Representative OAP traces at color asterisks labeled locations are presented. Since perfusion of voltage sensitive dye is not perfectly uniform from endocardium (ENDO) to epicardium (EPI), the ΔF amplitude map is non-uniform before HIFU ablation. (C) ΔF amplitude map is further normalized to its initial state (temporal average of first 4 ΔF amplitude maps). (D) Upon HIFU ablation, normalized ΔF amplitude decreased within HIFU focal region and led to OAPs amplitude reduction. The amount of normalized ΔF amplitude reduction is defined as ΔAPA and the reconstructed ΔAPA is illustrated with representative OAPs trace.
(DOC)

Figure S3 **Example of generating ultrasound parametric images.** (A) An example of ultrasound M2D-mode envelope image generated from ultrasound backscattered radio-frequency (RF) signals is presented with a red rectangular window (100×13 pixels). (B) Histogram of pixel intensity within highlighted window is plotted as blue bars. Envelope of the histogram is fitted using probability density functions (pdf) of Rayleigh (Rayl, green) and log-normal (Logn, red) distribution model. Rayleigh parameter *α* and log-normal scale parameter σ can be estimated from corresponding pdfs using maximum likelihood estimation method. (C) Rayleigh parametric image is reconstructed with green asterisk labeled pixel indicating *α* value within the highlighted window in (A). (D) Log-normal parametric image with red asterisk corresponding to the same window in (A). (E) Horizontally stacked gray-scale (GS) M2D-mode ultrasound images in decibels [dB] with blue dash lines representing the start and cessation of HIFU ablation. (F) Transient changes of GS images [dB] by subtracting adjacent frames in E. (G) Cumulative changes of GS images [dB] by subtracting all frames with the average of initial 4 – 5 frames. (H) Cumulative extrema of GS images computed by keeping the maximum absolute value of each pixel in the |ΔGS|_c_ images. Values of |ΔGS|_max_ are further normalized from 0 to unity.
(DOC)

Figure S4 **Example of lesion masks detection from wedge gross, histology, and triphenyltetrazolium stained images.** (A) A Photograph of a gross wedge within the field-of-view (FOV) of M2D-mode ultrasound imaging. (B) A binary lesion mask detected from the gross wedge image via an intensity threshold based k-means and Markov random field dilution algorithm. (C) A masson's trichrome (MT) stained slide of wedge from the same FOV in (A). (D) A binary lesion mask detected from the MT slides using a combined algorithm of image segmentation and edge detection. (E) A photograph of the same wedge in (A) after triphenyltetrazolium (TTC) staining. Necrotic tissue is stained as white while viable myocardium is stained as dark red. (F) Corresponding lesion binary mask detected from the TTC photograph via a color-intensity based algorithm.
(DOC)

Figure S5 **Activation maps with pacing performed at various locations relative to a HIFU lesion.** (A) Photograph of a canine left ventricle (LV) wedge preparation after HIFU lesion formation with epicardium (Epi) on the right and endocardium (Endo) on the left. HIFU lesion is indicated by the arrow. (B) Activation map superimposed on the LV wedge in (A) before HIFU ablation with pacing placed close to the base of the wedge. (C) Activation map superimposed on the LV wedge after HIFU ablation with pacing placed below the lesion and close to the base of the wedge. (D) Activation map superimposed on the same wedge after HIFU ablation with pacing placed in the center of lesion. No electrical activation was provoked from the pacing site whereas spontaneous activation was initiated from earlier pacing site. (E) Activation map superimposed on the same wedge after HIFU ablation with pacing at the endocardial side the lesion, resulting in a reformatted activation pattern. (F), Activation map superimposed on the same wedge after HIFU ablation with pacing above the lesion close to apex. Activation was re-established with origin at the pacing site. Pacing threshold was 2X or 3X of the threshold voltage (3V) for all experiments. Isochrones of activation map was separated with step size of 1 ms with blue representing early activation and red representing late activation. Missing areas on all activation maps are due to the placement of the pacing electrode which blocked the view of optical mapping.
(DOC)
