# Supplementary figures and images for: Transmural Ultrasound Imaging of Thermal Lesion and Action Potential Changes in Perfused Canine Cardiac Wedge Preparations by High Intensity Focused Ultrasound Ablation
Source: PLoS One. 2013 Dec 12;8(12):e82689. doi: 10.1371/journal.pone.0082689 (PMC3861459; doi:10.1371/journal.pone.0082689)

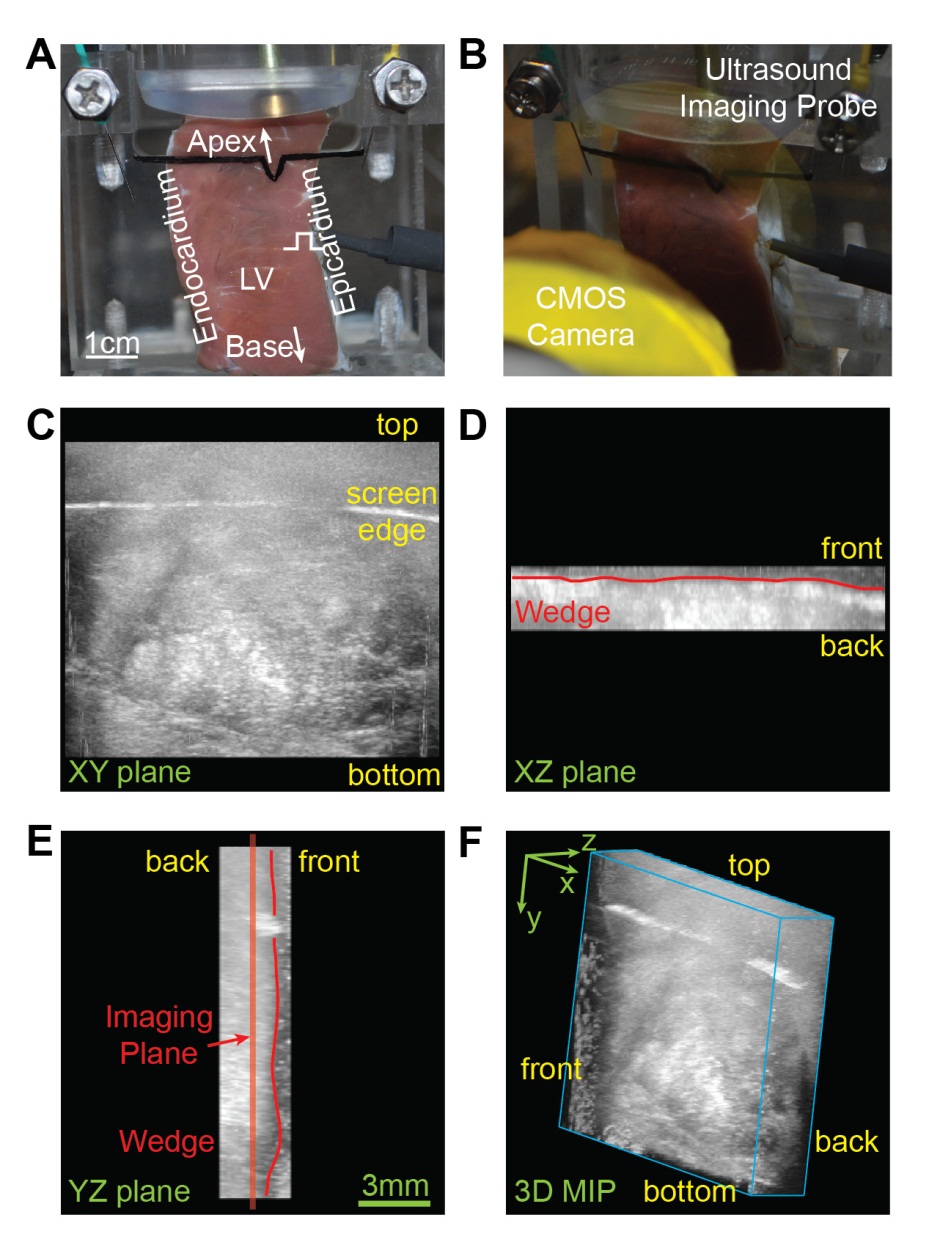

Supplement: Figure S1 — Relationship between optically mapped plane and ultrasound imaged plane. (A) A canine left ventricular (LV) wedge preparation was mounted on a custom tissue holder with the transmural surface gently pushed against an acoustic transparent polycarbonate screen. Pacing was applied from epicardium (right). (B) The transmural surface of the LV wedge is optically mapped as demonstrated. Emitted fluorescence signals beneath the surface (<700 µm) were detected by CMOS camera for optical mapping. High frequency ultrasound imaging was performed from the top window of the tissue holder. Ultrasound imaging plane was adjusted through a high precision XYZ motor (0.05 µm step-size) to ensure the field-of-view (FOV) close enough to the LV wedge transmural surface. (C) A sequence of ultrasound B-mode imaging with 0.2 mm step size was performed to form a 3D ultrasound volumetric image to verify ultrasound imaged plane. Ultrasound B-mode image of the frontal plane of the transmural surface is presented. The top edge of flat imaging screen is visible in B-mode image. (D) Transverse plane of 3D ultrasound imaging volume with a solid red line highlighting the transmural surface of the LV wedge. (E) Sagittal plane of the same LV wedge with a red solid line representing wedge transmural surface and an orange thick line indicating the on-line ultrasound imaging plane during experimentation. Ultrasound imaged plane is verified <0.7 mm below the wedge transmural surface. (F) 3D comprehensive view of ultrasound imaged volume. A bright hyperechoic region close to the top is the edge of the flat screen. (DOC) [file pone.0082689.s001.doc]

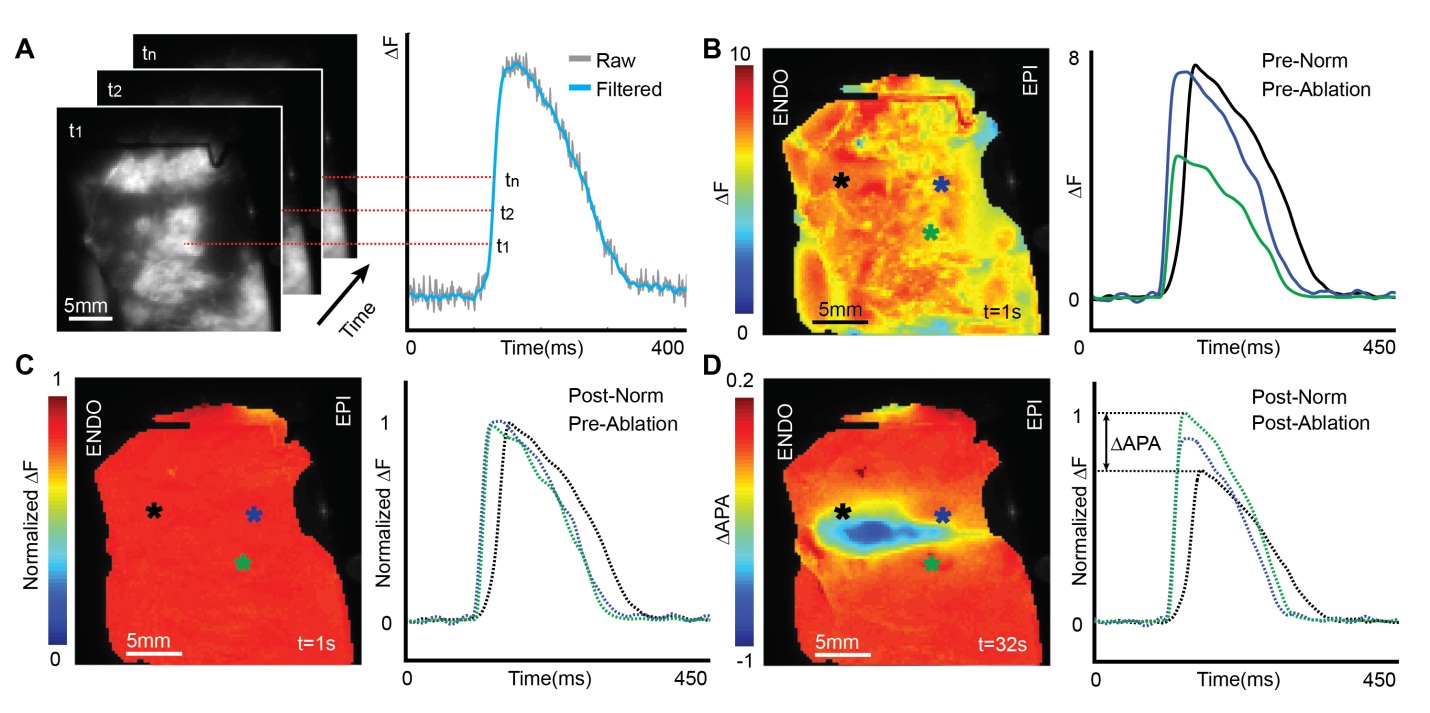

Supplement: Figure S2 — Example optical mapping data analysis. (A) Sequential images (t1, t2, …, tn) were acquired via optical mapping on a canine left ventricular (LV) wedge. Temporal recording at each pixel corresponds to optical transmembrane action potential. Raw optical signal is filtered through a 1 – 100 Hz band-pass FIR filter and fractional fluorescence changes (ΔF) is used to describe optical action potentials (OAPs). (B) Within one cardiac cycle, map of ΔF amplitude before high-intensity focused ultrasound (HIFU) ablation is generated. Representative OAP traces at color asterisks labeled locations are presented. Since perfusion of voltage sensitive dye is not perfectly uniform from endocardium (ENDO) to epicardium (EPI), the ΔF amplitude map is non-uniform before HIFU ablation. (C) ΔF amplitude map is further normalized to its initial state (temporal average of first 4 ΔF amplitude maps). (D) Upon HIFU ablation, normalized ΔF amplitude decreased within HIFU focal region and led to OAPs amplitude reduction. The amount of normalized ΔF amplitude reduction is defined as ΔAPA and the reconstructed ΔAPA is illustrated with representative OAPs trace. (DOC) [file pone.0082689.s002.doc]

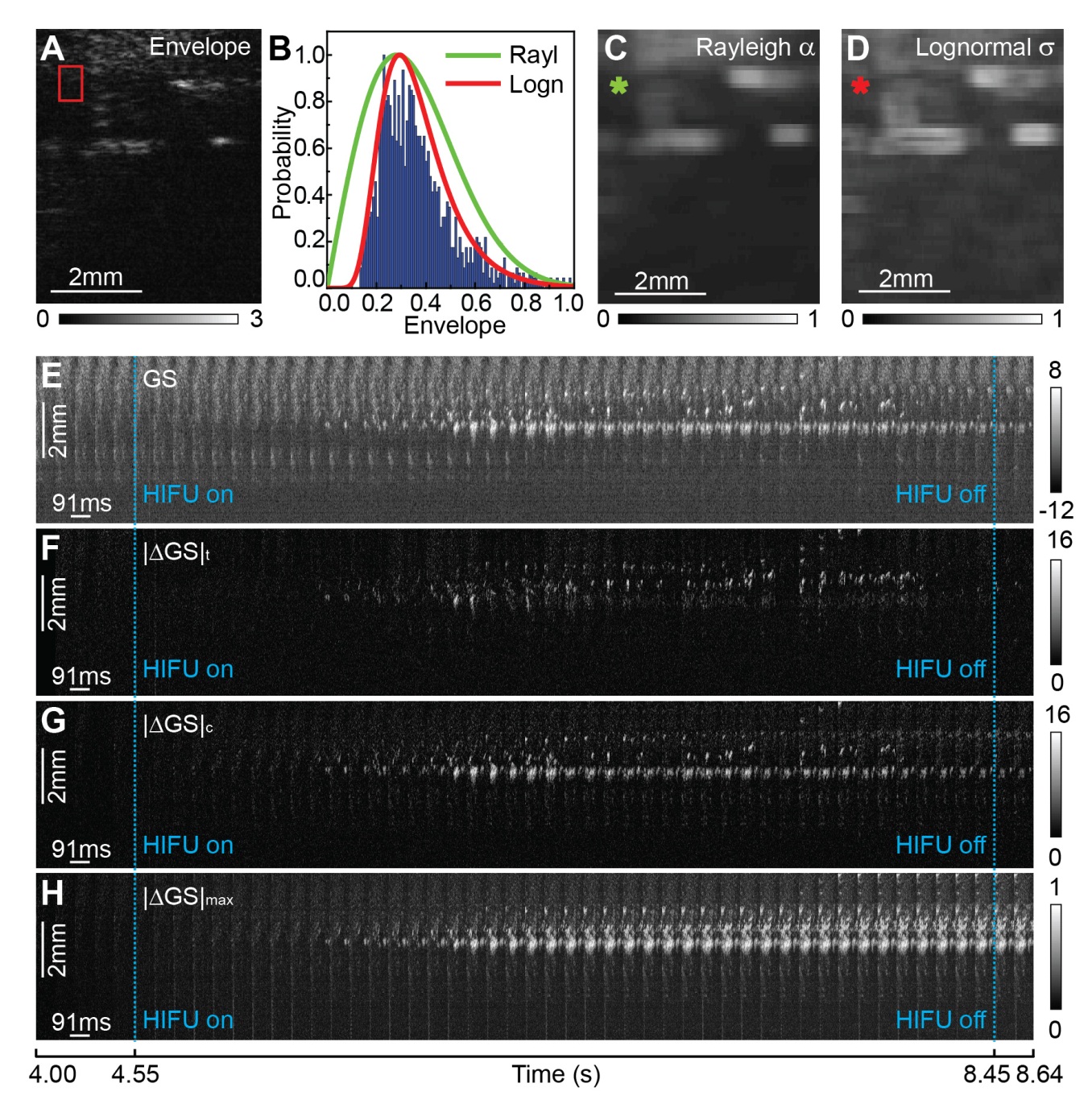

Supplement: Figure S3 — Example of generating ultrasound parametric images. (A) An example of ultrasound M2D-mode envelope image generated from ultrasound backscattered radio-frequency (RF) signals is presented with a red rectangular window (100×13 pixels). (B) Histogram of pixel intensity within highlighted window is plotted as blue bars. Envelope of the histogram is fitted using probability density functions (pdf) of Rayleigh (Rayl, green) and log-normal (Logn, red) distribution model. Rayleigh parameter α and log-normal scale parameter σ can be estimated from corresponding pdfs using maximum likelihood estimation method. (C) Rayleigh parametric image is reconstructed with green asterisk labeled pixel indicating α value within the highlighted window in (A). (D) Log-normal parametric image with red asterisk corresponding to the same window in (A). (E) Horizontally stacked gray-scale (GS) M2D-mode ultrasound images in decibels [dB] with blue dash lines representing the start and cessation of HIFU ablation. (F) Transient changes of GS images [dB] by subtracting adjacent frames in E. (G) Cumulative changes of GS images [dB] by subtracting all frames with the average of initial 4 – 5 frames. (H) Cumulative extrema of GS images computed by keeping the maximum absolute value of each pixel in the |ΔGS|c images. Values of |ΔGS|max are further normalized from 0 to unity. (DOC) [file pone.0082689.s003.doc]

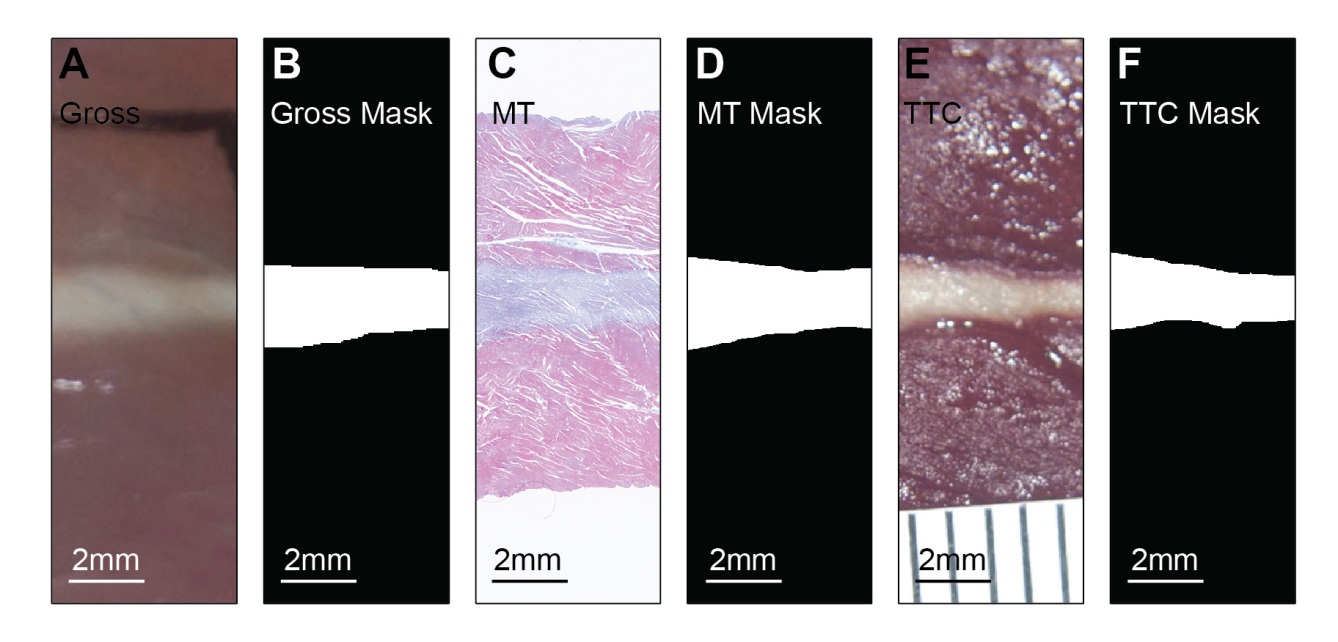

Supplement: Figure S4 — Example of lesion masks detection from wedge gross, histology, and triphenyltetrazolium stained images. (A) A Photograph of a gross wedge within the field-of-view (FOV) of M2D-mode ultrasound imaging. (B) A binary lesion mask detected from the gross wedge image via an intensity threshold based k-means and Markov random field dilution algorithm. (C) A masson's trichrome (MT) stained slide of wedge from the same FOV in (A). (D) A binary lesion mask detected from the MT slides using a combined algorithm of image segmentation and edge detection. (E) A photograph of the same wedge in (A) after triphenyltetrazolium (TTC) staining. Necrotic tissue is stained as white while viable myocardium is stained as dark red. (F) Corresponding lesion binary mask detected from the TTC photograph via a color-intensity based algorithm. (DOC) [file pone.0082689.s004.doc]

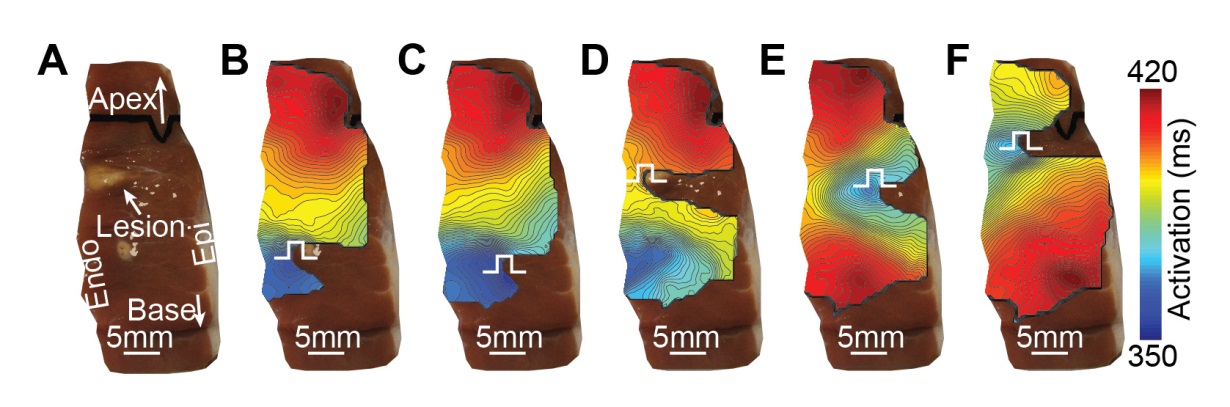

Supplement: Figure S5 — Activation maps with pacing performed at various locations relative to a HIFU lesion. (A) Photograph of a canine left ventricle (LV) wedge preparation after HIFU lesion formation with epicardium (Epi) on the right and endocardium (Endo) on the left. HIFU lesion is indicated by the arrow. (B) Activation map superimposed on the LV wedge in (A) before HIFU ablation with pacing placed close to the base of the wedge. (C) Activation map superimposed on the LV wedge after HIFU ablation with pacing placed below the lesion and close to the base of the wedge. (D) Activation map superimposed on the same wedge after HIFU ablation with pacing placed in the center of lesion. No electrical activation was provoked from the pacing site whereas spontaneous activation was initiated from earlier pacing site. (E) Activation map superimposed on the same wedge after HIFU ablation with pacing at the endocardial side the lesion, resulting in a reformatted activation pattern. (F), Activation map superimposed on the same wedge after HIFU ablation with pacing above the lesion close to apex. Activation was re-established with origin at the pacing site. Pacing threshold was 2X or 3X of the threshold voltage (3V) for all experiments. Isochrones of activation map was separated with step size of 1 ms with blue representing early activation and red representing late activation. Missing areas on all activation maps are due to the placement of the pacing electrode which blocked the view of optical mapping. (DOC) [file pone.0082689.s005.doc]
